# Prediction of local convergent shifts in evolutionary rates with *phyloConverge*

**DOI:** 10.1093/bioinformatics/btaf366

**Published:** 2025-07-16

**Authors:** Elysia Saputra, Weiguang Mao, Guillermo Hoffmann Meyer, Nathan Clark, Maria Chikina

**Affiliations:** Department of Computational and Systems Biology, Joint Carnegie Mellon University—University of Pittsburgh Program in Computational Biology, Pittsburgh, PA 15213, United States; Department of Computational and Systems Biology, University of Pittsburgh, Pittsburgh, PA 15213, United States; Department of Computational and Systems Biology, University of Pittsburgh, Pittsburgh, PA 15213, United States; Department of Biological Sciences, University of Pittsburgh, Pittsburgh, PA 15213, United States; Department of Biological Sciences, University of Pittsburgh, Pittsburgh, PA 15213, United States; Department of Computational and Systems Biology, University of Pittsburgh, Pittsburgh, PA 15213, United States

## Abstract

**Motivation:**

Convergence analysis can characterize genetic elements underlying morphological adaptations. However, its performance on regulatory elements is limited due to their modular composition of transcription factor motifs, which have rapid turnover and experience different evolutionary pressures.

**Results:**

We introduce *phyloConverge*, a phylogenetic method that performs scalable, fine-grained local convergence analysis of genomic elements at flexible length scales. Using a benchmarking case of convergent subterranean mammal adaptation, *phyloConverge* identifies rate-accelerated conserved noncoding elements (CNEs) with high specificity and statistical robustness relative to competing methods. From CNE-level scoring, we detect the convergent regression of entire CNE units and highlight the contrast that subterranean-associated coding region regression is highly specific to ocular functions, whereas regulatory element regression is enriched for accompanying neuronal phenotypes and other developmental processes. From transcription factor motif-level scoring, we dissect elements into subregions with uneven convergence signals and demonstrate the modular adaptation of CNEs with high functional specificity. Finally, we demonstrate *phyloConverge’*s scalability to perform high-resolution convergence analysis genome-wide.

**Availability and implementation:**

*phyloConverge* is available at https://github.com/ECSaputra/phyloConverge

## 1 Introduction

Decoding the genetic basis of complex phenotypes is a central goal of biology. Comparative genomics provides a valuable avenue for studying genotype–phenotype associations by using large-scale phenotypic diversity beyond what can be achieved with direct manipulation or observed at the population level. Genotype–phenotype mappings can be inferred by comparing the genomic sequences of species with an extreme phenotype with orthologous sequences in other species. Convergent evolution, whereby multiple species independently develop a trait, provides a statistical framework that allows us to distinguish phenotype-associated evolutionary processes from lineage- or species-specific changes.

To correlate genotypes and convergent traits, we focus on the evolutionary rate (ER) approach, which hypothesizes that when independent lineages convergently adapt to a common selection pressure, genetic elements that drive the selected phenotypes would undergo similar selective shifts. Elements under increased selective constraints would shift to a slower ER, while elements under relaxed constraints or positive selection would shift to a faster ER. The successful utility of this framework has produced several computational algorithms (Prudent *et al.* 2016, Hu *et al*. 2019, Kowalczyk *et al.* 2019).

These methods have demonstrated success in identifying genome-wide phenotypic associations for protein-coding and noncoding elements, but their application to noncoding regions is limited, because such methods require defined units of sequences to operate on. The typical strategy for defining noncoding units is to use PhastCons (Siepel *et al.* 2005), which segments the alignment into conserved regions. This approach produces a set of conserved noncoding elements (CNEs) that represent putative regulatory elements (REs) and have a size range of 50–500 bp, much larger than a single transcription factor binding site (TFBS). This disconnect between the CNE unit and the TFBS, which is the atomic unit of sequence activity, poses specific challenges for evolutionary analysis.

REs contain multiple TFBS, and dissection of well-characterized REs revealed a complex relationship between individual TFBS and functional output. Ablating TFBS may eliminate activity, change it, or have no effect (Serrano-Saiz *et al*. 2020, Snetkova *et al.* 2021). RE activity is itself multifactorial as many REs are pleiotropic, occurring via identical TFs binding to identical sites, different TFs binding to identical sites, and different site usage (Spivakov 2014). Individual TFBS can have context-specific contributions to regulatory activity and divergent evolutionary pressures. Thus, phenotype-driven changes in ERs may be more localized than typical CNE length, requiring a finer resolution evaluation.

Most alignment-based methods for convergence analysis correlate phenotype with measures of ER deviation from neutrality. The “Forward Genomics” branch method captures substitution rate shifts as sequence divergence between each pair of parent–child nodes (Prudent *et al.* 2016), while RERconverge uses maximum likelihood (ML) rate calculations with multiple statistical adjustments (Kowalczyk *et al.* 2019). However, these methods cannot efficiently scan alignments for local convergence signals, because they are designed to compute the signal for the entire unit of each given input alignment, with extensive preprocessing steps. For example, a unique phylogenetic tree must be computed for each local segment of an input alignment. Meanwhile, the *phyloP* framework in PHAST (Pollard *et al.* 2010) is based on the likelihood of a generative sequence model and thus can operate on very small alignments.

While *phyloP* is best known for scoring conservation by fitting a scaling parameter to a neutral substitution model, the framework is flexible and can fit additional clade- or branch set-specific scaling constants. We use the term *phyloP* (branch) to refer to the model with additional branch scaling. The result of adding an additional scaling parameter can be accessed statistically by the likelihood ratio test (LRT). However, the LRT may not produce well-behaved *P*-values because a function of a global neutral tree plus additional scaling parameters is an oversimplification. The most accurate model would give each branch its own scaling parameter to account for local variation. Consequently, increasing the number of parameters will often produce a significantly better fit even in the absence of a specific foreground signal. Previous findings also highlighted that *phyloP* cannot differentiate strong signals by a single branch versus from the convergence of multiple weaker branches (Hu *et al*. 2019), and that it is not sufficiently powerful for analyzing short segments (Pollard *et al.* 2010). Thus, while *phyloP* serves as a good foundation for building a scalable tool for performing large-scale scanning of REs, there is still a need to improve the robustness of the statistical predictions for accurately identifying true genotype–phenotype mappings.

Thus, we present *phyloConverge*, a fast comparative genomics method that performs fine-grained local convergence analysis to identify genomic regions associated with phenotypic convergence. Our method combines explicit parameterization of ER shifts and phylogeny-aware trait permutations to produce unbiased convergent rate shift scores calibrated to the local context of the chromosomal region.

## 2 Materials and methods

### 2.1 phyloConverge

**Figure 1.**
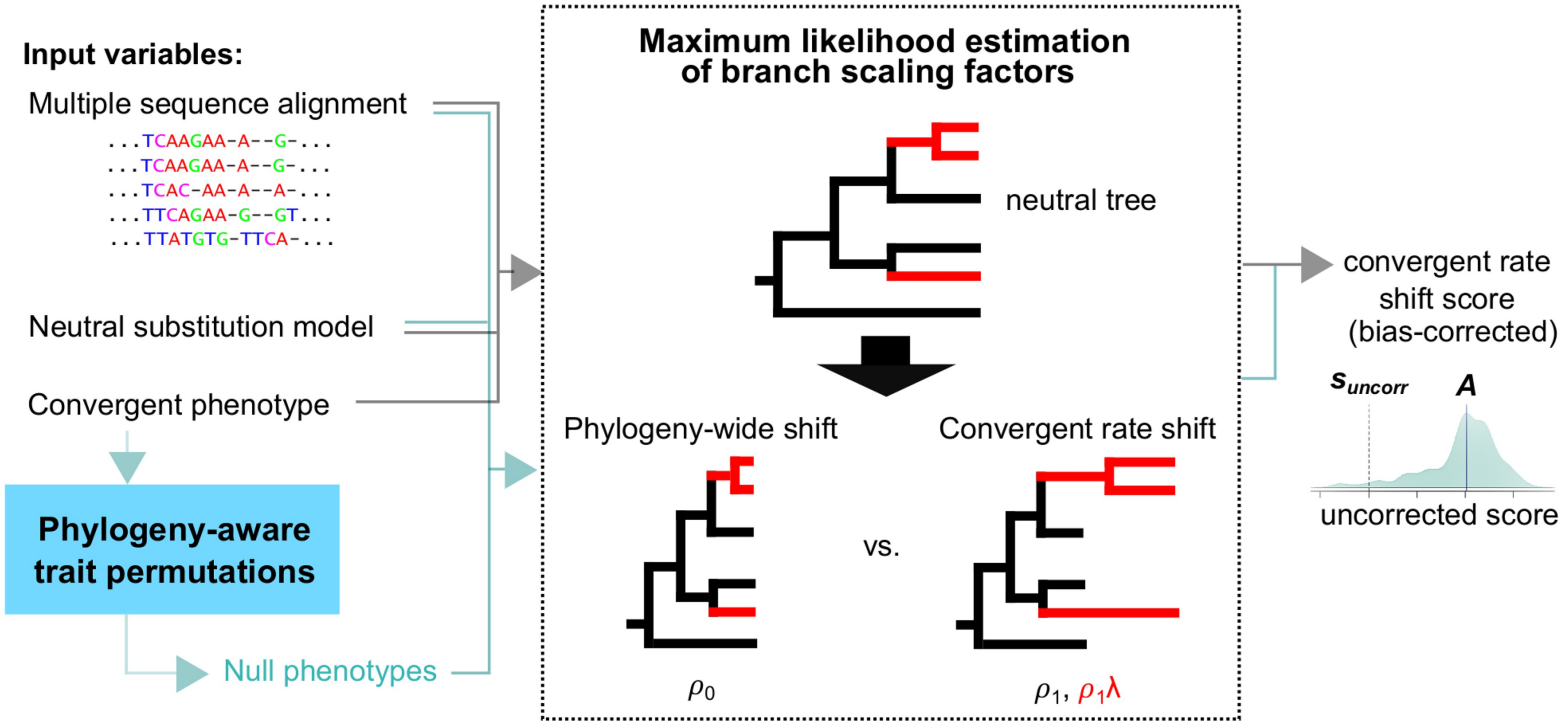
Workflow of *phyloConverge*. Given a set of species with convergent phenotype, a neutral model of evolution, and a multiple sequence alignment, *phyloConverge* combines generative nucleotide substitution modeling and phylogeny-aware trait permutation to compute convergent rate shift scores that are empirically corrected for statistical biases. Using maximum likelihood estimation of branch scaling factors, *phyloConverge* performs likelihood ratio test to test the null hypothesis that the foreground branches are not differentially scaled from the background branches, then conducts this test on numerous null phenotypes to correct for biases.

Given a multiple sequence alignment (MSA) of a region of interest, a phylogenetic model of neutral substitution, and a set of convergent species (i.e. “foregrounds”), *phyloConverge* computes the association between genetic changes and convergent phenotypic adaptation using a combination of ML estimation of ER shifts and phylogenetic statistical calibration. In *phyloP*, a convergent rate shift is inferred by performing ML estimation of two branch scaling factors (Fig. 1, Supplementary Methods S1.1, available as supplementary data at *Bioinformatics* online). The scaling factor ρ measures phylogeny-wide rate shifts relative to the neutral tree, and the scaling factor λ measures ER shifts that occur in the foregrounds. Evidence for rate convergence is quantified by an LRT comparing the null hypothesis of constant scaling across both foreground and background (i.e. all branches are uniformly scaled by $\rho_{o}$) against the alternative hypothesis that the foreground branches are scaled by λ in addition to the background scaling $\rho_{1}$. The convergence score is defined by computing the negative log-likelihood of the LRT *P*-value, noting the magnitude of λ.

To calibrate for biases, we previously developed a phylogenetic trait permutation method called permulation, a portmanteau of *permu*tation and simu*lation* (Saputra *et al*. 2021). Permulation produces “null” traits by selecting new sets of foregrounds that match the observed trait in the number of foregrounds and their phylogenetic dependence. Using Brownian motion simulations, permulation assigns phenotype values by taking a “random walk” down the phylogenetic tree, resulting in simulated values that are systematically more similar among closely related species. The properties of the null phenotypes are then determined by the rejection sampling (Supplementary Fig. S1, available as supplementary data at *Bioinformatics* online). With these null traits, we can perform permutation tests to correct the test statistics. We incorporate this trait permulation strategy into *phyloConverge* to produce *n* null traits and use *phyloP* (branch) to compute convergence scores for the observed and null traits (Supplementary Methods S1.2, available as supplementary data at *Bioinformatics* online). We measure the significance of rate shift by computing an empirical *P*-value $p_{\mathrm{corr}}$, defined as the proportion of the null *phyloP* scores that are as extreme or more extreme than the observed trait’s score. As the interpretation of the rate shift is bidirectional and the null distribution can be nontrivial, we calculated $p_{\mathrm{corr}}$ using the two-sided conditional *P*-value approach (Kulinskaya 2008), which transforms one-sided *P*-values into equivalent weighted two-sided *P*-values for symmetric or asymmetric distributions.

Although the permutation test is important for accurate calibration, a large number of computations are needed to achieve a high *P*-value resolution. Thus, we adopted an adaptive permutation strategy (Wang *et al.* 2019) that balances *P*-value resolution with running time by pruning the number of permutations if a target significance threshold has been crossed (Supplementary Methods S1.3, available as supplementary data at *Bioinformatics* online). This approach reduces the computational overhead greatly without incurring a loss in accuracy within the controlled significance level (Supplementary Fig. S2, available as supplementary data at *Bioinformatics* online). Suppose we aim to control for significance α with *N* permulations. Denoting $S^{\prime }$ as the set of computed null statistics, for a hypothesis to be statistically significant, the maximum number of extreme null statistics is αN, defined as the “pruning” threshold. After each permulation, we track whether this threshold has been reached, at which an adaptive $p_{\mathrm{corr}}$ is computed. Then, the convergent rate shift score $s_{\mathrm{corr}}$ is computed as the negative logarithm of $p_{\mathrm{corr}}$, signed by the direction of the convergent shift (positive for deceleration and negative for acceleration). *phyloConverge* also measures the robustness of the convergence signals using a leave-one-out approach, in which parametric scoring with *phyloP* is repeated by removing one foreground species in each repetition. Convergence signals are robust if removing one foreground species does not immediately erase or flip the signals.

### 2.2 Datasets

**Figure 2.**
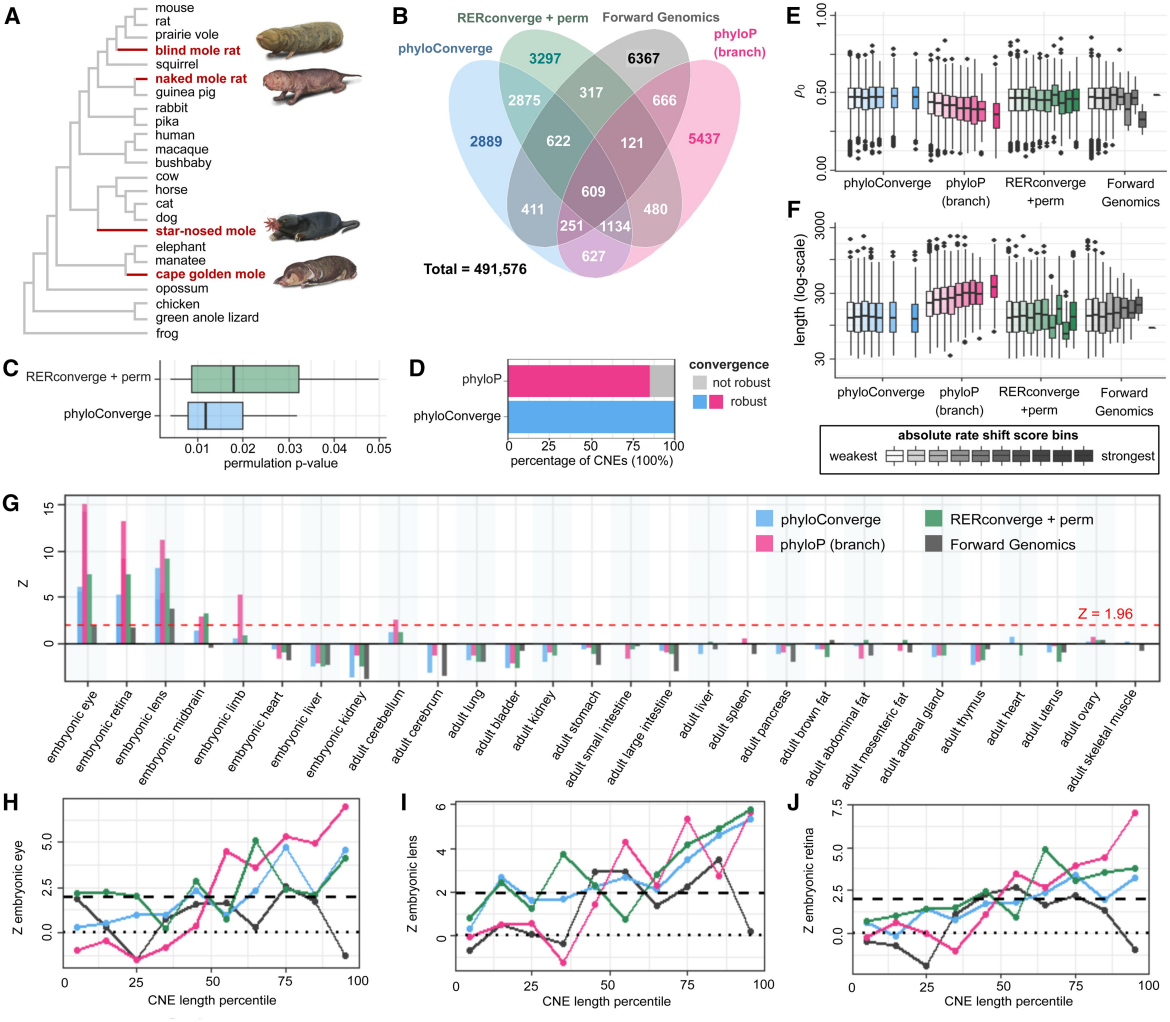
Benchmarking phyloConverge with the convergent evolution of conserved noncoding elements (CNEs) in subterranean mammals. (A) Phylogeny of benchmarking dataset. (B) Venn diagram showing overlaps between top ∼9400 subterranean-accelerated CNEs identified by phyloConverge, phyloP’s “branch” method, RERconverge+permulation, and Forward Genomics’s “branch” method. (C) Acceleration *P*-value distributions for the top ∼9400 CNEs from phyloConverge versus RERconverge+permulation. (D) Fraction of CNEs with robust versus nonrobust convergence signals as computed by phyloConverge versus phyloP (branch). (E) Correlations between conservation (smaller $\rho_{o}$) and the absolute values of convergence scores (grouped by equidistant score binning), and (F) the same for CNE lengths (in bp), among the top ∼9400 CNEs. (G) Functional enrichments for mouse tissue-specific open chromatin regions (OCRs) in subterranean-accelerated CNEs, plotted as *Z*-scores relative to 1000 null CNE sets. (H) Functional enrichments for mouse embryonic eye-specific OCRs in subterranean-accelerated CNEs binned based on length percentiles (0th–10th, 10th–20th, etc., dots representing the midpoints of the bins), (I) the same for mouse embryonic lens-specific OCRs and (J) mouse embryonic retina-specific OCRs.

As it is not understood how convergent rate shifts interact with other evolutionary factors, it is difficult to construct simulated data that reliably represent real data. Thus, we benchmark *phyloConverge* using a silver standard based on real data and a well-characterized convergent trait, the subterranean mammal habitat. We expect many of the convergent accelerations to be associated with degeneration of visual structures (Hetling *et al.* 2005). We used a dataset by Roscito *et al.* (2018), containing an MSA with a mouse (*mm10*) reference and 24 species, including four subterranean mammals (Fig. 2A) and 491 576 CNEs.

As validation datasets, we include retinal tissue-specific marker genes (Macosko *et al*. 2015), retinal cell-type-specific open chromatin regions (OCRs) identified from single-cell ATAC-seq data (Norrie *et al.* 2019), tissue-specific OCRs identified from bulk ATAC-seq data (Roscito *et al.* 2018, Liu *et al.* 2019, Gorkin *et al*. 2020) including ENCODE datasets (Luo *et al.* 2020) (identifiers: ENCFF251OST, ENCFF289KOI, ENCFF375KQJ), and a ChIP-seq dataset of OTX2 binding in human stem-cell derived retinal pigmented epithelium (Cohen-Gulkar *et al.* 2023) (GEO: GSE178165). We also used the position weight matrices of 771 TFBS motifs from the HOCOMOCO database (version 11) (Kulakovskiy *et al.* 2018) to predict TFBS coordinates genome-wide (Supplementary Methods S2, available as supplementary data at *Bioinformatics* online).

## 3 Results

### 3.1 Benchmarking phyloConverge on the convergent adaptation of subterranean mammals

We benchmark *phyloConverge* against three competing methods: *phyloP* (branch), RERconverge+permulation, and Forward Genomics branch method. Permulation was not applied to Forward Genomics, because the base method already applied phylogenetic bias correction (Prudent *et al.* 2016). As Roscito *et al.* (2018) identified 9364 “subterranean-accelerated” CNEs using Forward Genomics, we score the 491 576 CNEs using *phyloConverge*, *phyloP*, and RERconverge, and identify the top ∼9400 accelerated CNEs computed by each. Only 609 CNEs (∼6.5% of each set) are commonly identified by all methods (Fig. 2B). Notably, *phyloConverge* and RERconverge identify 5240 (∼55.5%) common CNEs, which is much higher than any other pair (17.6%–28%). While *phyloConverge* and RERconverge differ in their model framework, they both rely on ML estimates of ERs and permulation bias correction, suggesting that these features drive the observed overlap. However, we note that RERconverge underperforms in estimating substitution rates in short, local segments of CNEs, while *phyloP*, the baseline method of *phyloConverge*, resulted in healthy branch length distributions (Supplementary Fig. S4A, available as supplementary data at *Bioinformatics* online). Comparing the *P*-value distributions of accelerated CNEs from *phyloConverge* and RERconverge (Fig. 2C), which have the same method of empirical *P*-value generation, *phyloConverge* shows a better Type I error control. *phyloConverge* also has a similar run-time to RERconverge+permulation (Supplementary Fig. S4B, available as supplementary data at *Bioinformatics* online). Finally, we find robust convergence signals for 99.4% of the accelerated CNEs identified by *phyloConverge* compared to 84.5% for *phyloP* (Fig. 2D).

Next, we benchmark the methods for their specificity against confounders. A challenge in using comparative genomics to infer genotype–phenotype mapping is that the phenotype-unaware conservation signal is already highly associated with functional data. Thus, we evaluate the correlation between the scaling factor $\rho_{o}$ (phylogeny-wide conservation) and the absolute magnitude of convergence scores (Fig. 2E). *phyloP* indeed shows a negative correlation between $\rho_{o}$ and absolute convergence scores (see Supplementary Fig. S5, available as supplementary data at *Bioinformatics* online, for all CNEs), meaning that without statistical calibration, stronger convergence signals are given to more strongly conserved elements. Bias is also observed for Forward Genomics, which corrects for phylogenetic nonindependence by computing branch-specific sequence identity values (Prudent *et al.* 2016). The empirical correction approach utilized by *phyloConverge* and RERconverge completely removes this bias. We find a similar trend with longer regions being more likely to have strong convergence scores for *phyloP* and Forward Genomics (Fig. 2F). RERconverge and *phyloConverge* scores show uniform distributions across lengths, suggesting their potential for minimizing bias in scoring short TFBS-scale regions versus long CNE-scale regions. Overall, *phyloConverge* has superior statistical behaviors compared to competing methods.

To evaluate the specificity of biological inference, we compute the associations of accelerated CNEs with tissue-specific “marker” OCRs across mouse tissues. By tracking the overlaps between accelerated CNEs and marker OCRs, we observe that all methods produce strong enrichments (permulation *P*≤.05 or *Z* > 1.96) for OCRs of embryonic whole eye, retina, and lens, relative to 1000 randomly selected size-matched null CNE sets (Fig. 2G), which agrees with our expectation. For these eye tissues, *phyloConverge* produces strong signals that almost match RERconverge, and are much stronger than Forward Genomics. We also observe that not correcting for biases [*phyloP* (branch)] produces strong associations with tissues for which excessive relaxation of genetic elements is not expected, such as embryonic limb. Meanwhile, the remaining three methods show no excessive enrichment for marker OCRs of nonocular tissues for which enrichment is not expected.

We also benchmark the specificity of the methods for detecting ocular signals at different sequence lengths. We bin the CNEs according to lengths (0th–10th percentile, 10th–20th,…,90th–100th) and calculate the enrichment of the accelerated CNEs for ocular OCRs relative to other CNEs in the same length category (Fig. 2H–J). *phyloP* (branch) and Forward Genomics generally underperform at shorter lengths, but *phyloP*’s signals increase with length. Meanwhile, *phyloConverge* and *RERconverge* show similar performances with consistent positive enrichment values across sequence lengths, noting stronger enrichments (*Z*≥1.96 or permulation *P*≤.05) for longer sequences.

### 3.2 Subterranean-accelerated elements are enriched for distinct functions from accelerated coding regions

**Figure 3.**
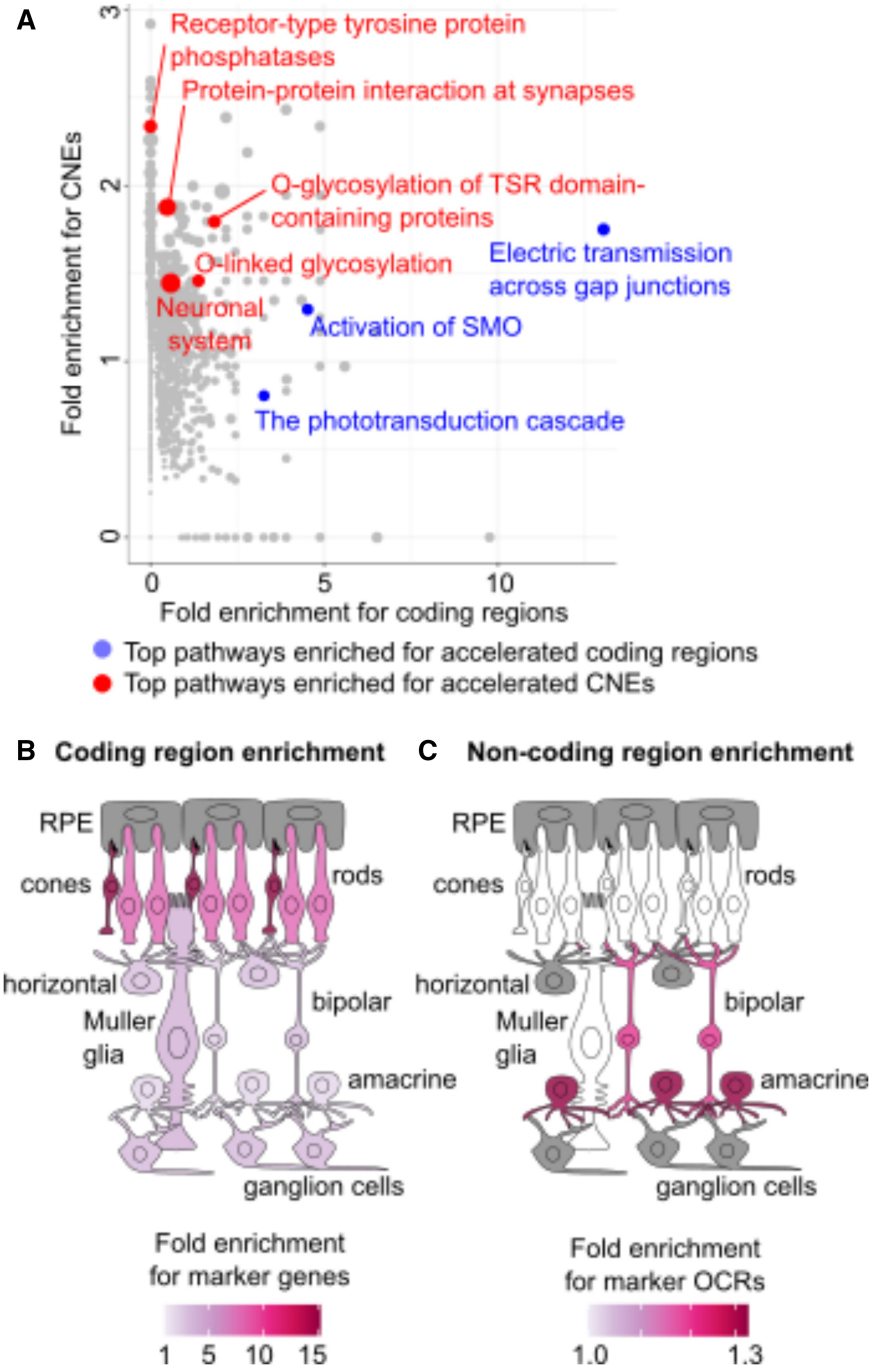
Coding region versus CNE acceleration occurs across distinct biological functions. (A) Contrasting the functional enrichment of coding and noncoding acceleration. (B) Accelerated coding regions show the strongest enrichment for cone and rod photoreceptor marker genes. (C) Accelerated CNEs are enriched for amacrine and bipolar cells. White: Benjamini–Hochberg adjusted enrichment *P* > .05; gray: missing genetic or genomic annotations.

We use GREAT (McLean *et al*. 2010) to evaluate the functional associations of the accelerated CNEs identified by *phyloConverge*. We also use *phyloConverge* to score 19 816 protein-coding regions and examine the pathway enrichments (Fig. 3A). The pathways enriched for accelerated coding regions are distinct from those for accelerated CNEs. The pathways enriched for accelerated coding regions include phototransduction, a vision-specific process, while those enriched for accelerated CNEs include neuronal functions.

A similar trend emerges when we examine enrichments for retinal cell-type-specific marker genes (Macosko *et al*. 2015) and OCRs (Norrie *et al.* 2019). The accelerated coding regions are enriched for all retinal cell types, but the cone and rod photoreceptors show drastically stronger enrichments (Fig. 3B). In contrast, accelerated CNEs are only enriched for amacrine and bipolar cells (Fig. 3C). Photoreceptors are specialized for phototransduction and express many specific genes dispensable for nonocular functions. Meanwhile, amacrine and bipolar cells are specialized interneurons that relay visual signals, and their expression profile is similar to other interneurons (Taylor and Smith 2012, Euler *et al.* 2014).

These observations are consistent with the known distinctions between the regulation of developmental versus tissue-specific genes. Enhancers regulating developmental genes are longer (Li and Wunderlich 2017) and located in large regulatory domains with multiple elements controlling different functions (Pachano *et al*. 2022), while genes active in differentiated cells are controlled by shorter enhancers proximal to the promoter. Retinal-specific genes have specific functions and simpler regulation. Thus, relaxation of pressure on ocular functions would largely act on coding regions. Meanwhile, neuronal cells can specialize into different cell types, and fate-committed neuronal cells still have plasticity driven by dynamically regulated transcriptional changes (Gallegos *et al.* 2018). Thus, in response to a relaxation of constraint on ocular functions, it is likely that selective pressure would still act on the coding regions, but a subset of enhancers involved in visual functions would experience acceleration.

### 3.3 Transcription factor motif-scale convergence signals associated with subterranean adaptation

**Figure 4.**
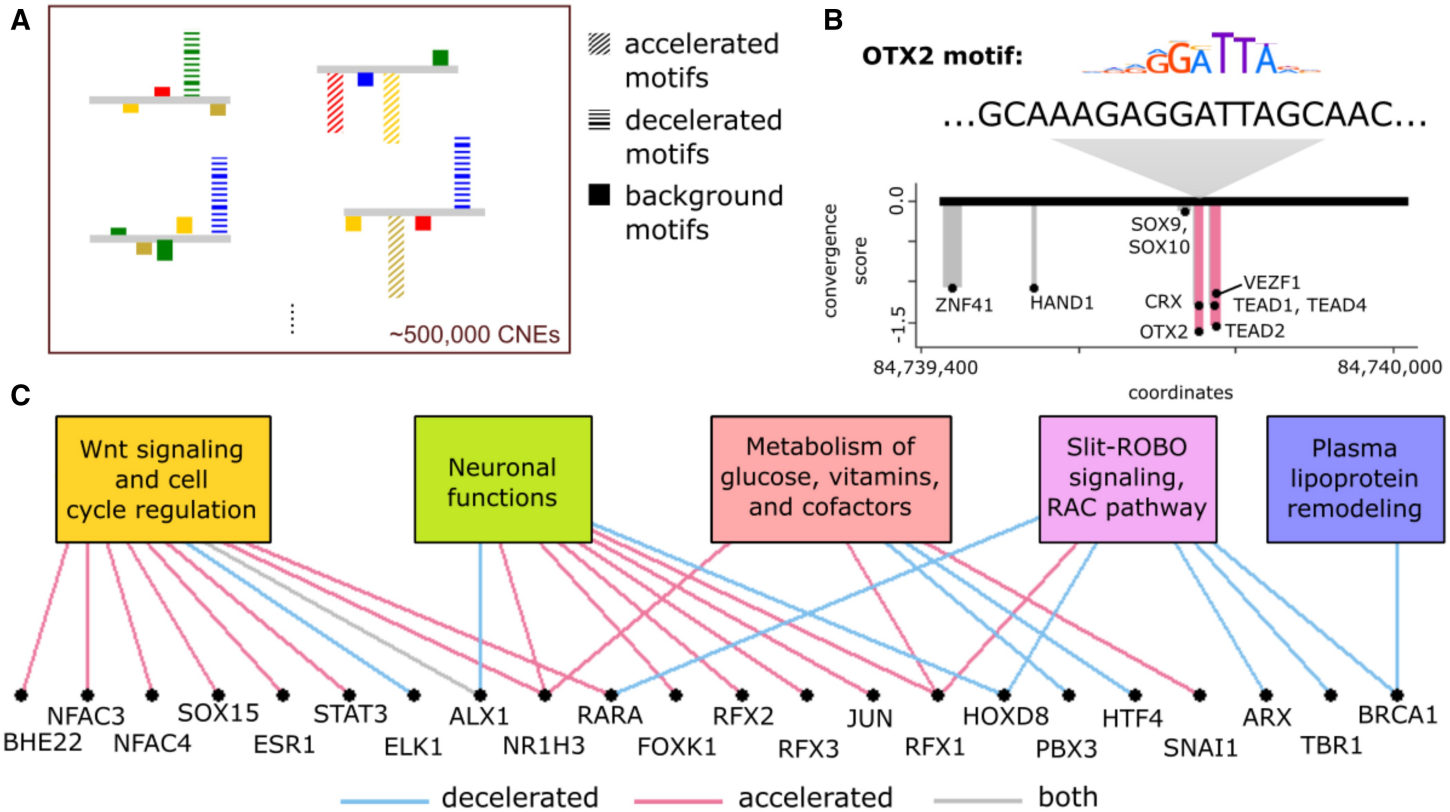
Transcription factor binding site (TFBS) motif-level convergence analysis reveals modular changes in CNEs underlying subterranean mammal adaptation. (A) TFBS motifs overlapping CNEs are scored for acceleration/deceleration. (B) CNE206119. (C) Functional categories enriched for motifs with global convergent signals.

The *phyloConverge* framework can fit the convergent rate shifts model at an arbitrarily small, even base-pair, resolution, allowing for a deeper investigation into the information content of different parts of a CNE. To understand how convergent shifts are reflected in TFBS profiles, we identify 1 761 185 TFBS matches within the 491 576 CNEs and compute their convergence scores (Fig. 4A). With an empirical *P*≤.05 and filtering for signal robustness, we find 42 477 significantly accelerated and 81 101 significantly decelerated motifs.

Local convergence scores can highlight the nonuniformity of evolutionary pressures acting on segments of an element, providing hypotheses that can be tested using functional assays. For example, Fig. 4B shows CNE206119, which overlaps an OTX2 binding site from a ChIP-seq dataset of human stem cell-derived retinal pigmented epithelium cells (Cohen-Gulkar *et al.* 2023). The segment of CNE206119 that shows the strongest acceleration indeed corresponds to the OTX2 consensus motif. CNEs can exhibit multiple sites of acceleration, deceleration, or a mix of both (Supplementary Fig. S6A–C, available as supplementary data at *Bioinformatics* online).

Additionally, we find motifs that show global convergent shifts in relation to subterranean adaptation. Specifically, we find significant enrichments for 43 motifs in the set of 42 477 accelerated motifs, and for 117 motifs in the 81 101 decelerated motifs, with 11 motifs showing global acceleration and deceleration (Supplementary Table S2, available as supplementary data at *Bioinformatics* online). Given the promiscuity of TFs, we ask if these signals are mainly concentrated on specific relevant functions. We use GREAT to perform motif-specific functional enrichment analysis for the acceleration/deceleration of each motif, given the genome-wide distribution of the given motif. With false discovery rate (FDR)≤0.05, we identify the enrichment for 43 unique and interrelated pathways across 22 motifs (Supplementary Table S3, available as supplementary data at *Bioinformatics* online). For example, RFX3 plays a role in multiple functions, including neuronal, cardiac, and ion homeostasis, but the acceleration of RFX3 motifs in subterranean mammals is strongly specific to neuronal functions only. This suggests that the functional specificity of phenotypic adaptation can be modularly mediated by convergent shifts of binding sites located near relevant genes. Organizing the enriched annotations based on their correlations, the annotations mainly comprise five categories, including “Wnt signalling and cell cycle regulation,” “neuronal functions,” “metabolism of glucose, vitamins and cofactors,” “Slit-ROBO signaling and RAC pathway,” and “plasma lipoprotein remodeling” (Fig. 4C).

## 4 Conclusion

*phyloConverge* offers the scalability to perform rapid, calibrated scoring at flexible resolution. Although permulation incurs additional computational cost, the adaptive termination strategy confers a significant speedup because complete permulation would only be performed for elements with significant phenotypic associations, which only constitute a small fraction. *phyloConverge* can be extended to perform unsupervised genome-wide scans for convergence, generating convergence tracks similar to the *phyloP* conservation score without prior definitions of functional units. While we provide an example approach in Supplementary Text S1, available as supplementary data at *Bioinformatics* online, as the conservation patterns of noncoding regions can be nontrivial, future work should evaluate how rate shifts in these regions should be interpreted.

Finally, predictions from alignment-based methods should be interpreted with caveats. Enhancers can have homologous functions across distantly related species despite lacking sequence conservation (Wong *et al.* 2020). Prediction also relies on the global alignability of sequences, which deteriorates with increasing evolutionary distance. “Alignment-free” methods comparing sequences in a functional readout space may be appropriate for these cases, but alignment-based methods would be sufficiently powerful for strongly conserved promoter regions and developmental enhancers.

## Supplementary Material

btaf366_Supplementary_Data

## Data Availability

The alignment dataset was published in Roscito *et al.* (2018) and available at https://bds.mpi-cbg.de/hillerlab/CNEDivergence/. Other datasets are publicly available in the cited publications and repositories. Supplementary data and functions can be found at https://github.com/ECSaputra/phyloConverge-manuscript. *phyloConverge* is available at https://github.com/ECSaputra/phyloConverge with DOI: 10.5281/zenodo.15330765.
