# Supplementary material for: Prediction of local convergent shifts in evolutionary rates with phyloConverge
Source: Bioinformatics. 2025 Jul 16;41(7):btaf366. doi: 10.1093/bioinformatics/btaf366 (PMC12275462; doi:10.1093/bioinformatics/btaf366)
Supplement: btaf366_Supplementary_Data [file btaf366_supplementary_data.pdf]

Supplementary Data

Supplementary Data S1: *phyloConverge* scores for conserved non-coding elements (CNEs)

Supplementary Data S2: *phyloConverge* scores for coding regions

Supplementary Data S3: *phyloConverge* scores for transcription factor binding sites intersecting CNEs

Supplementary Methods

S1. *phyloConverge*

phyloConverge computes the association between convergent genetic changes and convergent phenotypic adaptation using a combination of maximum likelihood-based estimation of evolutionary rate shifts and phylogenetic statistical calibration of test statistics. The input of *phyloConverge* includes a multiple sequence alignment (MSA), a phylogenetic model of neutral nucleotide substitution (which can be estimated from sites that are expected to undergo neutral evolution, e.g., fourfold-degenerate sites), and the list of species with the convergent phenotype.

S1.1. Generative modeling of convergent evolutionary rate shifts with *phyloP*

To quantify the uncorrected association score between the evolutionary rate of a genetic element and the convergent phenotype, *phyloConverge* uses the *phyloP* function in the RPHAST package[1,2]. Given the neutral nucleotide substitution rates and branch lengths in the phylogenetic model computed from neutral sites, *phyloP* performs maximum likelihood estimation to scale the branch lengths in the model to optimize the probability of observing the given MSA. The null hypothesis–that the foreground (convergent) branches are not differentially scaled from the rest of the branches–requires the estimation of one phylogeny-wide branch scaling factor ρ_o. The alternative hypothesis–that the foreground branches are differentially scaled from the rest of the branches–requires the estimation of two scaling factors: a phylogeny-wide scaling factor ρ_1 and a foreground-specific scaling factor λ. *phyloP* then performs a hypothesis test to determine whether the null model or the alternative model better fits the observed MSA. *phyloConverge* uses the likelihood ratio test (LRT) for the hypothesis testing and adopts the 'CONACC' scoring method defined by *phyloP* (positive scores denote deceleration, negative scores denote acceleration).

S1.2. Empirical calibration with phylogenetic permulation

To empirically calibrate for statistical biases, *phyloConverge* performs permutation tests by producing numerous null or 'fake' phenotypes and using *phyloP* to compute null scores from them. To generate the null phenotypes, we previously developed a phylogeny-aware strategy for permuting phenotype trees that is grounded on phylogenetic simulation, called permulation[3]. The objective of the permulation strategy is to generate permutations that capture the existing structure in the data. Permulation achieves this by preserving the phylogenetic features in the observed phenotype, which in binary traits are captured by the number of foreground species and the phylogenetic dependence between the foreground species. First, the method performs a Brownian motion phylogenetic simulation based on the given trait tree and neutral tree. Next, the simulated branch length values are used to inform the selection of a new set of branches that will be proposed as a candidate null phenotype, specifically by choosing the branches with top-ranking branch length values. A rejection sampling process is then employed to determine if the candidate null phenotype would be accepted as valid, in which two conditions must be met: that the null phenotype has the same number of foreground branches, and that the foreground branches have matching phylogenetic relationship as observed in the true

phenotype. After numerous valid null phenotypes are obtained, *phyloP* is used to compute scores for each of the null phenotypes, such that a null distribution of *phyloP* scores for the given MSA is obtained. We previously developed two permulation strategies for binary phenotypes: the 'complete case' (CC) method, which produces null phenotype trees from the complete topology, and the 'species subset match' (SSM) method, which accounts for missing sequences in a particular MSA. While the SSM method is more stable and accurate, the CC method is significantly faster, with comparable if slightly less accuracy. For tractability, *phyloConverge* currently makes use of the CC method.

In such a permutation test, the significance of deviations from the expected value is typically measured by computing empirical p-values that are defined as the proportion of the null statistics that are as extreme or more extreme than the observed test statistic. In a two-tailed test, these extreme values make up the area under the curve beyond the observed statistic and the negative of the observed statistic. As *phyloP* defines acceleration as a negative score and conservation as a positive score following the 'CONACC' scoring mode, the two tails of the null score distribution signify opposing directions of rate shift, where the lower tail denotes acceleration and the upper tail denotes deceleration. Because of this directionality and because the null distribution is not necessarily trivial or symmetric, we calculated the two-tailed empirical p-value p_{corr} using the two-sided conditional p-value approach described by Kulinskaya[4], which transforms one-sided p-values into equivalent, weighted two-sided p-values for symmetric or asymmetric distributions. Suppose the distribution of null *phyloP* scores follows a strictly increasing continuous cumulative distribution function F. Then, p_{corr} is computed as follows:

$$p_{corr} = \frac{F(s_{uncorr})}{F(A)} \mathbb{I}[s_{uncorr} \leq A] + \frac{1-F(s_{uncorr})}{1-F(A)} \mathbb{I}[s_{uncorr} \geq A],$$

where s_{uncorr} is the uncorrected score (computed by *phyloP*) for the observed phenotype and A is the value that the null distribution is centered on. For our purposes, A was chosen as the median of the null scores, such that the weights at both the left and right sides of A were equal. Subsequently, the bias-corrected conservation/acceleration score s_{corr} is computed as the negative logarithm of p_{corr}, signed by the relative position of s_{uncorr} with respect to A, as follows:

$$s_{corr} = -\log_{10} p_{corr} \operatorname{sign}(s_{uncorr} - A).$$

S1.3. Adaptive permulations

To improve the computational tractability of permulations, we incorporated a simple strategy to adaptively terminate permulations when a target significance threshold had been reached. For example, suppose we would like to control for significance threshold $\alpha = 0.05$ with a maximum of 1000 permutations. For a genetic element to be significantly associated with the convergent trait, there can only be a maximum of 50 null scores that are as extreme or more extreme than the observed uncorrected score. Formally, suppose we want to control the test for a significance level of α, and we set a maximum of N permulations. Denoting S' as the set of computed null statistics, for a hypothesis to be statistically significant at α significance level, the maximum number of null statistics that are as extreme or more extreme than the true statistic s_{uncorr} is therefore αN, defined as the "pruning" threshold. At every permulation iteration i, we track whether the pruning threshold has been reached, given the value of the median of the null distribution at iteration i, A_i. If the threshold has been reached, the adaptive p_{corr} is computed as follows:

$$p_{corr} = \frac{\min\left(\alpha N+1,\ \sum_{s' \in S'} \mathbb{I}[s' \leq s_{uncorr}]\mathbb{I}[s_{uncorr} \leq A_i] + \mathbb{I}[s' \geq s_{uncorr}]\mathbb{I}[s_{uncorr} > A_i] + 1\right)}{\min\left(N+1,\ \sum_{s' \in S'} \mathbb{I}[s' \leq A_i]\mathbb{I}[s_{uncorr} \leq A_i] + \mathbb{I}[s' \geq A_i]\mathbb{I}[s_{uncorr} > A_i] + 1\right)}.$$

The addition of '+1' to each term is done to correct the tail ends of the distribution. The approach indeed offers remarkable improvements in speed -- parallelizing over 60 cores on one compute node with 95GB memory, the scoring of ~36,000 CNEs with 500 permulations can be completed in ~1.5 hours. Using ~5,000 randomly selected subset of the CNEs dataset and the subterranean foregrounds, the empirical p-values calculated from all 500 permulations (p_{total}) and the adaptive

empirical p-values (p_{corr}) computed to control significance levels α of 0.05 correlate very well (Pearson's R = 0.978, p-value < 2.22 x 10-16), with negligible loss in resolution within the significance level that is controlled (Supplementary Fig. S1). However, we note the necessity for weighing the trade-off between the maximum number of permulations and the α level to control. For example, with a maximum of 500 permulations, setting $\alpha = 0.01$ means that only a maximum of 5 extreme null scores are allowed such that the computations may be prematurely terminated. In such cases, the performance of adaptive permulation may suffer, because stochasticity can cause premature termination of the permulations. The R implementation of *phyloConverge* is available on GitHub (https://github.com/ECSaputra/phyloConverge).

S2. Transcription factor binding site (TFBS) motif calling

Genome-wide scanning for possible TFBS motifs was performed using PWMScan[5]. The parameters for PWMScan include the genome assembly of interest, the position weight matrix (PWM) of the motif of interest, and a threshold cutoff for calling the TFBS motif. We obtained the PWMs of 771 TFBS motifs from the HOCOMOCO database (version 11)[6]. We then used *motifDiverge*[7] to compute the background frequency of each nucleotide based on the probability matrix of a given TFBS and infer the PWM matrix of each TFBS and the TFBS calling cutoff to use (set to control Type I error rate below 10^{-5}). Using these input parameters, we previously made the genome-wide TFBS calls for other uses with the human *hg19* coordinate from the UCSC Genome Browser[8]. These genome-wide calls were then lifted over to the mouse *mm10* coordinate using the liftOver tool from UCSC[9]. Finally, the set of conserved TFBS motifs were identified by intersecting the TFBS coordinates with the CNE coordinates using BEDtools.

S3. Identification of tissue-specific "marker" open chromatin regions (OCRs)

To evaluate the functional enrichments of top-ranking subterranean-accelerated CNEs, we computed the correlations between the CNEs with tissue-specific, "marker" open chromatin regions (OCRs) in mouse tissues. For mouse embryonic tissues, we compiled publicly available ATAC-seq datasets (see Supplementary Table S1 for identifiers). The marker OCRs for the whole eye, retina, and lens were taken directly from Supplementary Data 16 of Roscito et al.[10]. For the remaining tissues, the datasets with multiple replicates were first pre-processed by identifying consensus regions (regions that were present across at least 2 replicates) using the *GenomicRanges*[11] package in R. Subsequently, the marker OCRs of each given tissue were obtained by subtracting regions that were open in any other tissue from the tissue of interest using BEDTools[12].

We also used the chromatin accessibility atlas across adult mouse tissues[13]. Given that the dataset was presented in the format of consensus peaks, we first identified the OCRs in each tissue by setting the 80th percentile of the read count distribution as a threshold. Then, we identified the marker OCRs of each given tissue by subtracting regions that were open in at least 80% of the other tissues. Finally, the regions were lifted over from the *mm9* to the *mm10* coordinates.

S4. Benchmarking *phyloConverge* against existing methods

We first used *phyloConverge*, *phyloP*, and RERconverge+permulation to compute the convergence scores of the set of CNEs produced by Roscito et al. For *phyloConverge* and RERconverge+permulation, 500 null phenotypes were used. For each method, the top ~9,400 CNEs were identified by selecting the appropriate threshold that would result in a set with a comparable size to the set produced by Roscito et al., specifically 9,377 CNEs with p-value ≤ 0.032 for *phyloConverge*, 9,455 CNEs with p-value ≤ 0.05 for RERconverge, and 9,325 CNEs with $s_{uncorr} \leq -5.1$ for *phyloP*. For the coding region analysis with *phyloConverge*, the same threshold of p-value ≤ 0.032 was used to select the top-ranking coding regions.

We applied the random subsampling strategy previously used by Roscito et al. to compute correlations between subterranean-accelerated CNEs with marker OCRs. Before computing correlations, we merged nearby subterranean-accelerated CNEs that were within 50bp apart to correct for inflation of significance resulting from multiple CNEs that were very close together. Afterwards,

for each tissue, we used BEDTools to find the number of intersections between the marker OCRs of the tissue and the subterranean-accelerated CNEs. We then subsampled 1,000 matched-sized sets of randomly selected CNEs from the total set of CNEs and similarly found the number of intersections with the marker OCRs to obtain the null distribution. The strength of correlation between the subterranean-accelerated CNEs and the marker OCRs were quantified as the Z-score computed with respect to the null distribution. This analysis was performed for the top-ranking subterranean-accelerated CNEs from the four methods tested.

To quantify the agreement between two sets of top-ranking subterranean-accelerated CNEs identified by two different comparative methods, we first noted the number of overlapping CNEs between the two sets using BEDtools. Then, we subsampled two sets of randomly selected CNEs from the total set, containing matching numbers of CNEs as the two actual sets, and similarly noted the number of overlapping CNEs. We performed the random subsampling 1,000 times to obtain a null distribution of the number of overlapping CNEs between two randomly selected sets of CNEs with the given sizes. The actual number of overlaps was then converted to a Z-score with respect to the null distribution.

S5. Functional enrichment analysis

To associate subterranean-accelerated CNEs with genes, we used the Genomic Regions Enrichment of Annotations Tool (GREAT)[14], specifically with the *rGREAT*[15] tool in R. We used GREAT's "twoClosest" setting to associate each CNE with the two TSS that are the closest to it. GREAT also defines the 'regulatory region' of each gene with proximal or distal CNEs using a default association rule called 'basal-with-extension'. GREAT captures proximal CNE associations by determining a 'basal regulatory region' around each gene, defined as the window within 1kb downstream and 5kb upstream of the transcription start site (TSS). Then, to capture distal associations, the regulatory domain of the gene is extended until it overlaps the basal regulatory region of neighboring genes, up to 1Mb both upstream and downstream. Afterwards, using the set of subterranean-accelerated CNEs as the 'foreground regions' and the total CNEs as the 'background regions', GREAT performs two statistical tests: (1) the hypergeometric test to compute the enrichment for foreground CNEs in each gene's regulatory domain, relative to the superset of background CNEs, and (2) the binomial test to compute the significance of observing a certain number of foreground CNEs in the regulatory domain given the proportion of all foreground CNEs over all CNEs. We used GREAT to perform all enrichment analyses for CNEs and motifs in this work, using Gene Ontology (GO) and Reactome pathway annotations. Significant hits were identified by setting a Benjamini-Hochberg adjusted p-value ≤ 0.05 for both tests, unless stated otherwise. For gene hits, GREAT only performed the hypergeometric test, and the significant threshold was set as Benjamini-Hochberg adjusted hypergeometric p-value ≤ 0.05.

We also performed enrichment analysis for the coding regions using the GO and Reactome pathway annotations. Fisher's exact test was used to compute the probability of observing the number of accelerated coding regions and background coding regions that overlapped members of a geneset given the total number of accelerated coding regions. The significance cutoff was set as Fisher's exact p-value ≤ 0.05.

S6. Enrichment analysis on retinal cell-type-specific marker genes and marker OCRs

We performed enrichment analysis on the top-ranking genes that were subterranean-accelerated in the coding regions using the retinal tissue-specific marker genes produced in Macosko et al.[16] as validation datasets. To perform enrichment analysis on the top-ranking subterranean-accelerated CNEs, we used a dataset of single cell ATAC-seq regions across different retinal tissues by Norrie et al.[17]. Clustering of single cells was performed using Seurat[18] and Signac[19] for the single cell RNA-seq and single cell ATAC-seq data, respectively, and tissue type assignments were made by integrating the multimodal datasets and transferring the single cell RNA-seq cluster labels to the corresponding single cell ATAC-seq clusters[20]. Cell type-specific marker OCRs were finally defined by finding the differentially accessible ATAC-seq peaks for the five resulting clusters (rods, cones, bipolar cells, amacrine cells, Müller glia). For both the coding and non-coding analysis, enrichment analysis was performed using the hypergeometric test. The cutoff for significant enrichment was set as Benjamini-Hochberg adjusted hypergeometric p-value ≤ 0.05.

S7. Transcription factor binding site (TFBS) motif-level convergence analysis

We used *phyloConverge* to compute convergence scores for individual conserved TFBS motifs that overlapped CNEs. Setting permulation p-value threshold ≤ 0.05 and with leave-one-out filtering, we identified 42,477 significantly accelerated motifs and 81,101 significantly decelerated motifs. To identify CNEs that underwent significant changes in motif content due to selection pressures ("motif-enriched"), we performed enrichment analysis to identify 3 categories of CNEs: (1) CNEs that were significantly enriched for accelerated motifs ("motif-accelerated CNEs"), (2) CNEs that were significantly enriched for decelerated motifs ("motif-decelerated CNEs"), and (3) CNEs that were significantly accelerated for both accelerated and decelerated motifs ("mixed-motif CNEs"). Enrichment analysis was performed using Fisher's exact test. Specifically, if we define the foreground motifs to be (1) the significantly accelerated motifs for the motif-accelerated category, (2) the significantly decelerated motifs for the motif-decelerated category, and (3) the significantly accelerated and decelerated motifs for the mixed-motif category, we tested the probability of observing m foreground motifs out of the n motifs in a CNE, given the total number of foreground motifs and the total number of conserved motifs. The significance threshold was set as Fisher's p-value ≤ 0.05. After the sets of motif-enriched CNEs were identified, functional enrichment analysis for each CNE set was performed using the GREAT tool. We used the Gene Ontology Biological Process, Cell Component, and Molecular Functions annotations, and significantly enriched annotations were identified by setting FDR ≤ 0.1 for both the binomial test and the hypergeometric test performed by GREAT.

To identify TFs whose binding motifs exhibit global convergence signals, we used Fisher's exact test to compute the enrichment for a given TF in the set of 42,477 significantly accelerated motifs and the set of 81,101 significantly decelerated motifs, respectively. For example, to compute the enrichment for TFBS A in the set of all significantly accelerated motifs, we computed the probability of observing n_A significantly accelerated motif A out of the 42,477 significantly accelerated motifs, given that there were a total of N_A TFBS calls for motif A and N_T total TFBS calls genome-wide. Motifs with global convergence signals were identified by setting Fisher's p-value ≤ 0.05. Pathway enrichment analysis of the motifs with global acceleration/deceleration signal was performed using GREAT, specifically using the Reactome pathway annotations. Significantly enriched annotations were identified by setting FDR ≤ 0.05 for both the binomial test and the hypergeometric test.

Finally, motif-specific functional enrichment analysis was also performed using GREAT with the Reactome pathway annotations, with FDR ≤ 0.05 for both the binomial and hypergeometric tests. Correlations between significantly enriched annotations were identified by empirically computing the probability of observing n number of overlapping genes between a pair of annotations, relative to the null distribution between a randomly selected pair of gene sets with matching sizes to the annotations of interest. The significance threshold for the correlations was set as empirical p-value ≤ 0.05.

Supplementary Text 1

Unsupervised scanning of conserved elements predicts segments with potential association with subterranean phenotype

In this section, we use phyloConverge to make predictions of phenotype-relevant TFBS-scale segments without requiring users to supply prior definition of known TFBS coordinates to score. We use phyloConverge to scan each nucleotide in the CNEs and compute the score from a window of ±5bp around the nucleotide, considering that ~10bp is the approximate scale of TFBS motifs. We find that the scanning output can highlight strongly accelerated and decelerated segments that correspond to known TFBS motifs. The top of Figure S8A shows the example scanning output for CNE327067, which is located close to SLC24A2, a cation/calcium ion exchanger that maintains the homeostasis of sodium, potassium, and calcium ion levels in the brain, retinal ganglion cells, and the retinal cone photoreceptors. We can see that the segment with the strongest acceleration signal corresponds to a known TFBS for POU4F2, a canonical retinal marker whose expression together with ISL1 has been found to be sufficient for specifying the retinal ganglion cell fate. We also observe that there are other strongly accelerated or decelerated segments that do not correspond to known TFBS motifs.

After performing the scanning on all the CNEs, we identified nucleotide segments that exhibit significant acceleration or deceleration signals. Out of the identified segments, only about 5.4% of them overlap with known TFBS coordinates that we previously called (Figure S8B). We then perform de novo motif discovery analysis for the newly identified segments, using the STREME tool. Setting an E-value threshold of 0.5, we found enrichment for 5 motifs in the new accelerated segments (Figure S8C), and 10 motifs in the new decelerated segments (Figure S8D). We used the TomTom motif comparison tool to test whether the enriched motifs are similar to known consensus motifs. Using an E-value threshold of 1 for the TomTom test, we characterize the known motifs that are significantly similar to the enriched motifs (Tables S4 and S5). Interestingly, there are 4 motifs that are enriched in both the accelerated and decelerated new segments. Some of these hits are associated with hypoxia response; FOXD2 (motif 5 in the decelerated set and motif 2 in the accelerated set) have been previously found to be enriched in the binding sites of HIF-2α (Hypoxia Inducible Factor 2 alpha) in HepG2 cells, SP1 /2 (motif 4 in the decelerated set and motif 5 in the accelerated set) are found in the binding sites of HIF-1α in RCC4 and HKC-8 cells, and STAT4 (motif 3 in the decelerated set and motif 1 in the accelerated set) are upregulated in the primary human macrophages under hypoxia. Another motif that is also enriched in both the accelerated and decelerated sets is SOX10, which is highly expressed in the brain. During development, SOX10 is expressed exclusively in oligodendrocyte precursor cells and is critical for controlling the maturation of oligodendrocytes. These findings further characterize the regulatory changes in neuronal development and hypoxia response that occur with subterranean adaptation.

Supplementary Figures

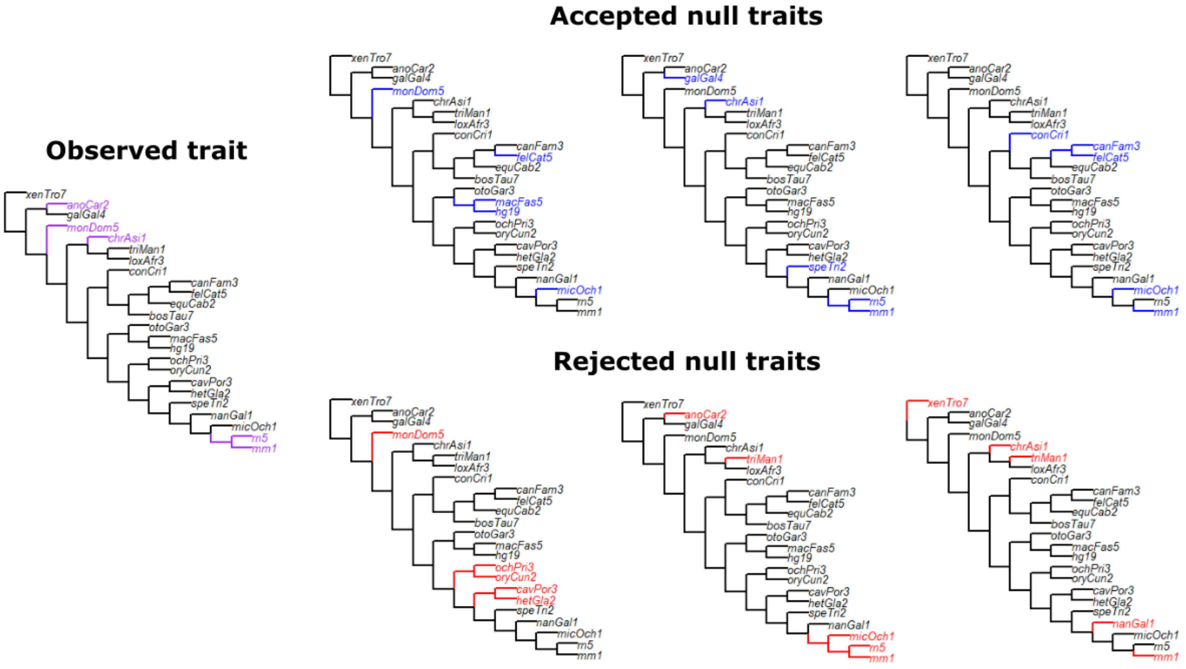

Supplementary Figure S1. Example null phenotypes produced by Brownian motion simulations and the outcome of rejection sampling in phylogenetic permulations. In this example, Brownian motion simulations are used to simulate phenotype values by performing a ‘random walk’ down the phylogenetic tree. The tip branches with top-ranking phenotype values, matching the number of the observed tip foregrounds, are proposed as potential null tip foregrounds. The potential null phenotype is rejected if the phylogenetic structure among the tip branches when shared ancestry is considered does not match the observed phylogenetic structure.

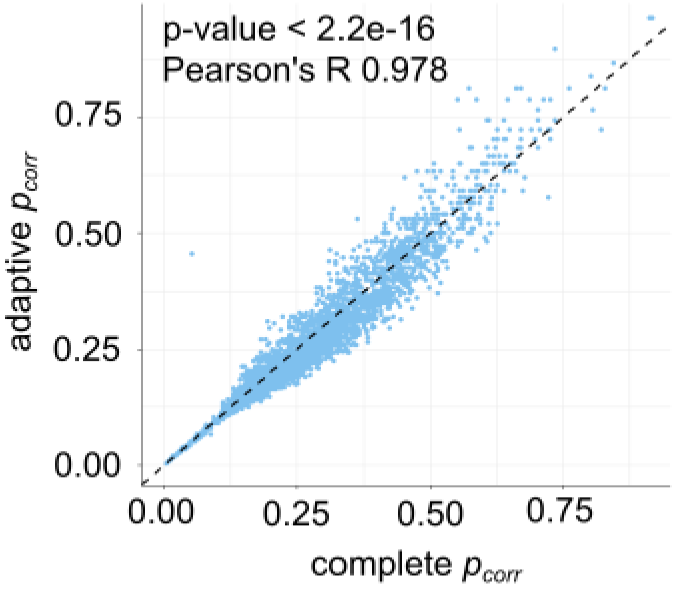

Supplementary Figure S2. Correlation between empirical p-values computed with adaptive permulations versus the complete permulations, with maximum permulations and controlling for significance level α of 0.05.

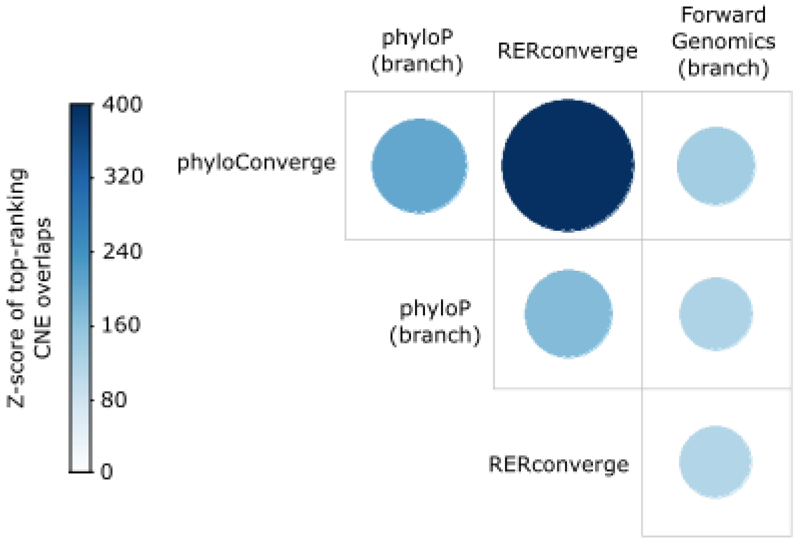

Supplementary Figure S3. Overlaps between top-ranking subterranean-accelerated conserved non-coding elements (CNEs) identified by *phyloConverge*, *phyloP* (branch), RERconverge, and Forward Genomics (branch) show very strong statistical significance. For each pair of sets of top-ranking CNEs from two different methods, the number of overlapping CNEs was first measured. Then, the total CNE set was subsampled twice to produce two sets of randomly selected CNEs containing matching numbers of CNEs as the two actual sets, and the number of overlapping CNEs between the two sets was measured. Performing the subsampling 1,000 times, the null distribution of the number of overlapping CNEs between two randomly selected sets of CNEs with the given set sizes was obtained, from which a Z-score could be computed. Notably, the Z-scores for all pairs were very strong (with *phyloConverge* and RERconverge showing the strongest statistical significance), with p-value ≈ 0.

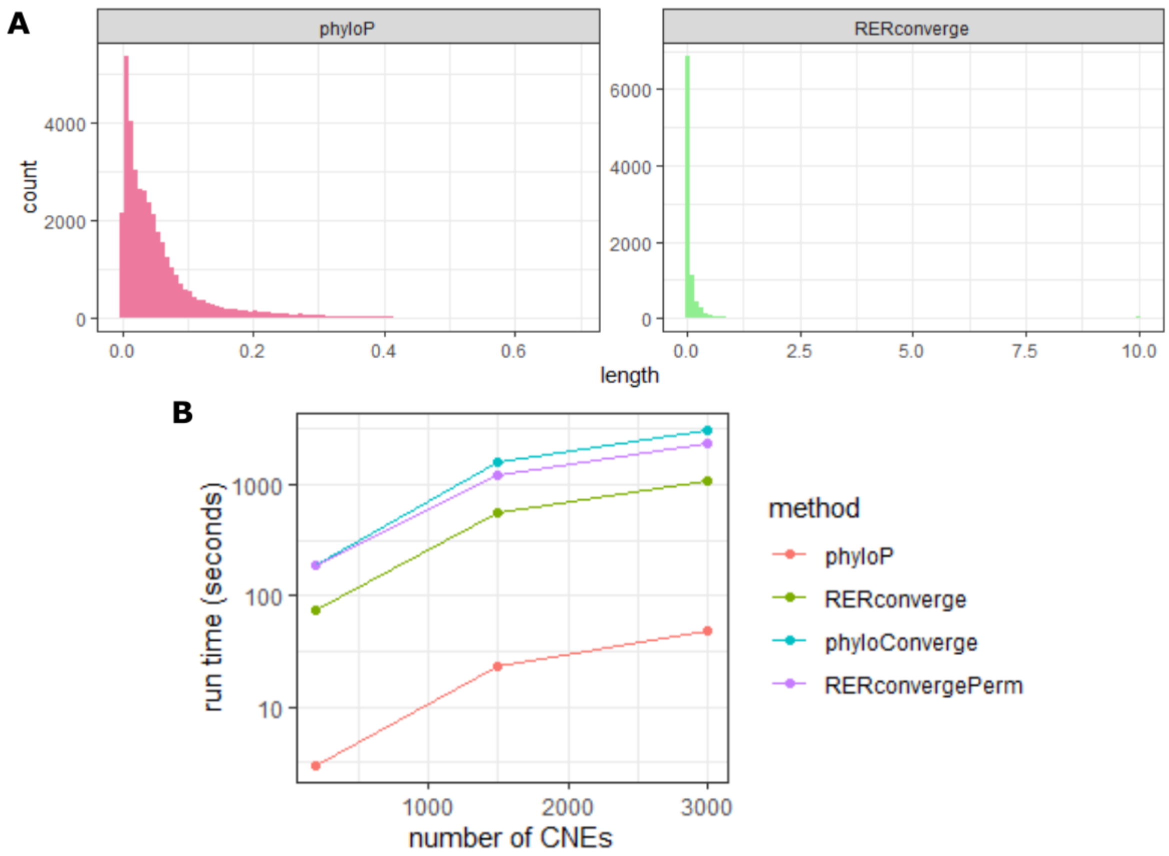

Supplementary Figure S4. (A) Distribution of estimated branch lengths of 10bp segments in 50 randomly sampled CNEs (sliding window of 10bp, stride 10bp). RERconverge resulted in branch lengths of ~0 for ~75% of branches in the segments, and tree estimation failed for segments in 35/50 CNEs. (B) Run time of *phyloConverge* compared to different methods on randomly sampled CNEs. For a fair comparison, all analysis were started with input files that included the alignment files of the CNEs, the convergent phenotype information, and a pre-computed neutral tree models as required by each method (computed from fourfold-degenerate sites for *phyloP* and *phyloConverge*, averaged from the phylogenetic tree of all ~500,000 CNEs for RERconverge-based methods). For methods that uses permutations, 1000 permulated phenotypes were used.

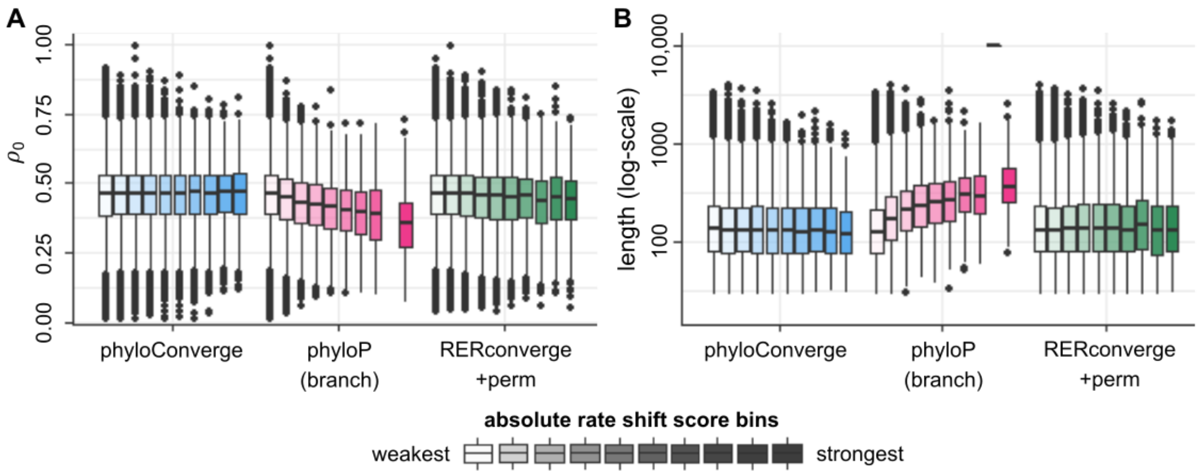

Supplementary Figure S5. (**A**) Correlations between conservation (smaller ρ_o) and the absolute values of rate shift scores (grouped by equidistant score binning), and (**B**) the same plot for CNE lengths (in bp), across all CNEs. Missing boxplots are due to different framework's ways of discretizing extreme values. Forward Genomics results for all CNEs were not publicly available.

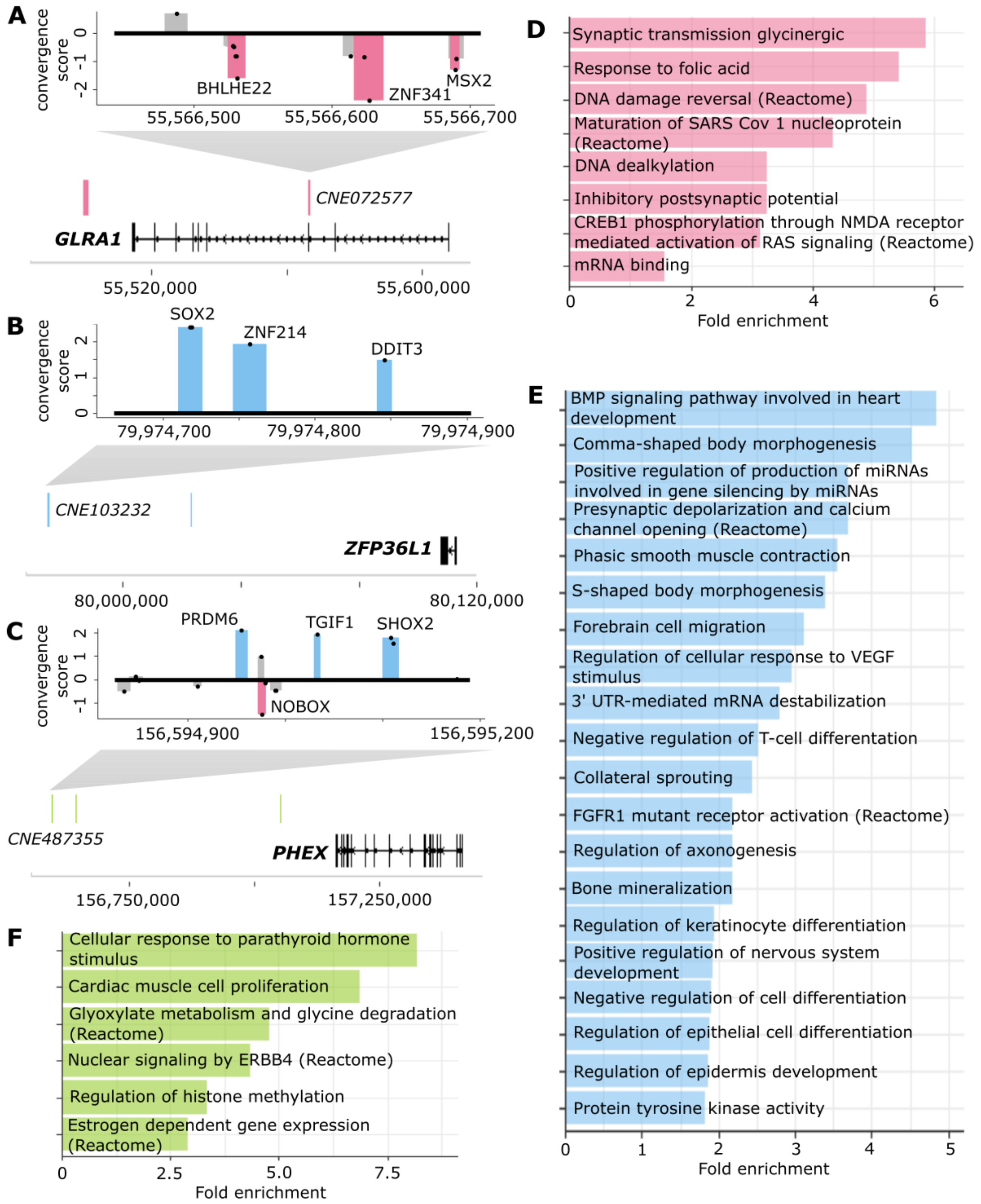

Supplementary Figure S6. Local convergence signals of transcription factor binding site (TFBS) motifs in a CNE highlight modularity of CNE function. (A) CNE072577 is enriched for significantly accelerated motifs. **(B)** CNE103232 is enriched for significantly decelerated motifs. **(C)** CNE487355 is enriched for significantly accelerated and decelerated motifs. **(D)** Gene Ontology and Reactome pathway terms that are associated with CNEs enriched for significantly accelerated motifs, **(E)** significantly decelerated motifs, and **(F)** both significantly accelerated and decelerated motifs.

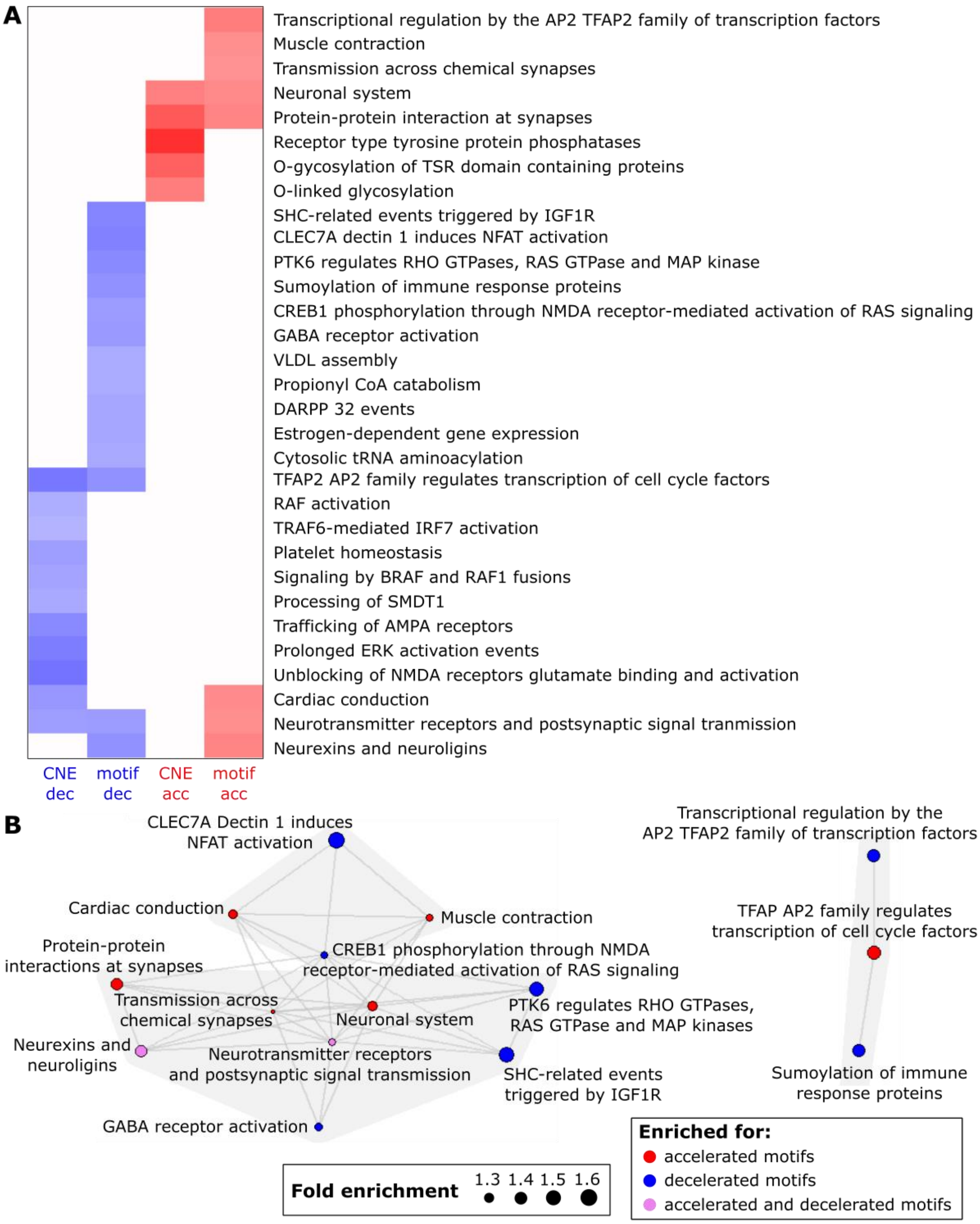

Supplementary Figure S7. (A) Reactome pathway annotations significantly enriched for accelerated or decelerated motifs and CNEs genome-wide. (B) Correlation structure among Reactome pathway annotations significantly enriched for accelerated or decelerated motifs.

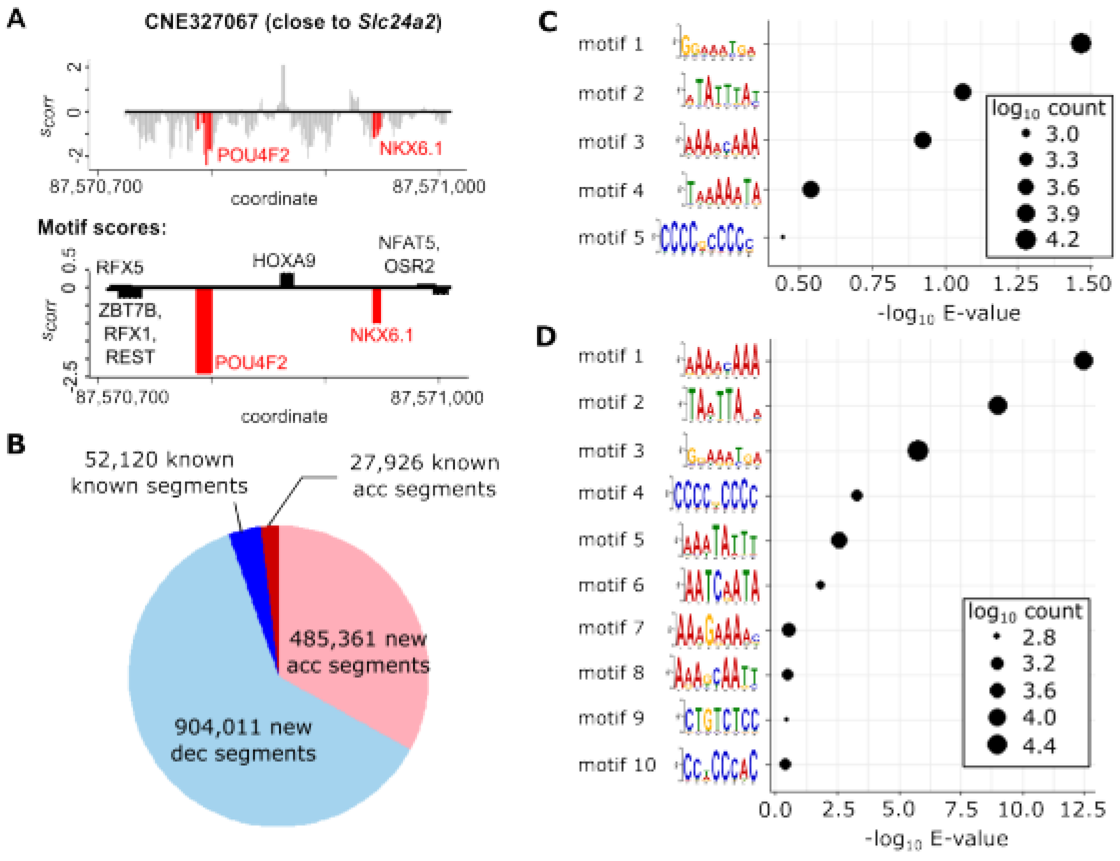

Supplementary Figure S8: Unsupervised scanning of CNEs proposes potential motifs undergoing significant convergent changes in subterranean adaptation. (A) The scanning output of an example CNE, CNE327067, where a sliding window of 11bp is used to compute the convergence signals at every nucleotide position. The bottom plot shows the corresponding scores for known TFBS motif coordinates. (B) The number of identified segments with significant convergent rate shifts from scanning. (C) De novo motif discovery analysis on accelerated segments that are newly identified without prior knowledge of motif coordinates, and (D) the same analysis for newly identified decelerated segments.

Supplementary Tables

Supplementary Table S1. List of publicly available datasets used for validation.

Dataset	Source
Mouse embryonic ATAC-seq, whole eye E11.5	Roscito et al.[10] (Supplementary Data 16)
Mouse embryonic ATAC-seq, retina E14.5	Roscito et al.[10] (Supplementary Data 16)
Mouse embryonic ATAC-seq, lens E14.5	Roscito et al.[10] (Supplementary Data 16)
Mouse embryonic ATAC-seq, midbrain E11.5	Roscito et al.[10]
Mouse embryonic ATAC-seq, limb E11.5	Roscito et al.[10]
Mouse embryonic ATAC-seq, kidney E14.5	ENCODE (Identifier: ENCFF251OST)
Mouse embryonic ATAC-seq, liver E14.5	ENCODE (Identifier: ENCFF289KOI)
Mouse embryonic ATAC-seq, heart E14.5	ENCODE (Identifier: ENCFF375KQJ)
Adult mouse ATAC-seq	Liu et al.[13]<br>Count matrix obtained from:<br>https://doi.org/10.6084/m9.figshare.c.4436264.v1
Mouse retinal single cell ATAC-seq	Norrie et al.[17]<br>scATAC-seq GEO accession number: GSM4995565<br>scRNA-seq GEO accession number: GSE164044
Retinal tissue marker genes	Macosko et al.[16] (Table S4)

Supplementary Table S2. Transcription factor motifs that are globally associated with subterranean adaptation.

Category	Motifs (HOCOMOCO identifier)
Accelerated	ALX1.0.B, ARX.0.D, ATF2.1.B, BHE41.0.D, CEBPB.0.A, COT2.0.A, DLX6.0.D, ERR2.0.A, ERR3.0.B, ETV7.0.D, FOXD1.0.D, GATA2.0.A, GLI1.0.D, HIC1.0.C, HSF4.0.D, HXD8.0.D, JUND.0.A, MEIS2.0.B, MYOD1.0.A, MYOD1.1.A, NFIB.0.D, NFIC.1.A, NKX61.1.B, NR4A3.0.D, RAX2.0.D, RFX1.1.B, RFX2.1.A, SALL4.0.B, SMAD3.0.B, SOX2.0.A, SOX9.0.B, TBX3.0.C, TFEB.0.C, THA.1.D, THB.0.C, UNC4.0.D, VENTX.0.D, Z324A.0.C, ZKSC3.0.D, ZN260.0.C, ZN317.0.C, ZN341.1.C, ZNF41.0.C
Decelerated	ASCL2.0.D, ATOH1.0.B, BACH2.0.A, BHA15.0.B, BHE22.0.D, BRCA1.0.D, DLX2.0.D, DLX5.0.D, ELK1.0.B, ERR2.0.A, ERR3.0.B, ESR1.1.A, ESR2.1.A, ETV4.0.B, ETV6.0.D, ETV7.0.D, FIGLA.0.D, FOS.0.A, FOSB.0.A, FOXJ2.0.C, FOXK1.0.A, FOXO4.0.C, FOXP1.0.A, FOXP2.0.C, FOXP3.0.D, GLI1.0.D, HAND1.1.D, HMGA1.0.D, HMX3.0.D, HSF1.1.A, HTF4.0.A, HXA9.0.B, HXB4.0.B, HXD4.0.D, IKZF1.0.C, ITF2.0.C, JUN.0.A, JUNB.0.A, JUND.0.A, KAISO.1.A, KLF13.0.D, KLF4.0.A, LYL1.0.A, MAF.1.B, MAFG.1.A, MECP2.0.C, MEF2D.0.A, MESP1.0.D, MSX2.0.D, MYF6.0.C, MYOD1.1.A, NDF1.0.A, NF2L1.0.C, NFAC1.1.B, NFAC2.0.B, NFAC3.0.B, NFAC4.0.C, NFIA.1.D, NFIB.0.D, NFIC.1.A, NFKB1.0.A, NFKB2.0.B, NGN2.0.D, NKX22.0.D, NKX25.0.B, NKX28.0.C, NOBOX.0.C, NR1H3.1.B, NR1H4.1.B, NR1I2.1.D, NR1I3.1.D, NR4A1.0.A, NR4A2.0.C, NR4A3.0.D, OLIG2.1.B, PBX1.0.A, PBX3.0.A, PBX3.1.A, PDX1.1.A, PHX2A.0.D, PO6F2.0.D, PPARA.1.B, PPARG.1.A, PRRX1.0.D, PRRX2.0.C, PTF1A.1.B, RARA.1.A, RARG.1.B, RFX1.1.B, RFX3.0.B, RFX4.0.D, RUNX3.0.A, RXRA.1.A, RXRB.0.C, SCRT1.0.D, SNAI1.0.C, SNAI2.0.A, SOX13.0.D, SOX15.0.D, SOX9.1.B, STA5A.0.A, STAT3.0.A, STAT4.0.A, SUH.0.A, TBR1.0.D, TFAP4.0.A, TFE2.0.A, TGIF1.0.A, THA.1.D, TWST1.1.A, UBIP1.0.D, ZBED1.0.D, ZBT18.0.C, ZEB1.0.A, ZN335.1.A, ZN554.1.D, ZN652.0.D"

Supplementary Table S3. Motif-specific pathway enrichment analysis results for transcription factor motifs that are globally associated with subterranean adaptation. Motif names are in HOCOMOCO identifiers.

**RFX1.1.B**<br>Accelerated		
**Reactome pathway ID**	**Hypergeometric fold enrichment**	**# gene hits**
Metabolism of water soluble vitamins and cofactors	5.80054	5
Regulation of expression of Slits and Robos	5.220486	5
Trafficking of AMPA receptors	5.568519	4
Nonsense mediated decay NMD	5.568519	4
Signaling by Robo Receptors	3.915365	6
Metabolism of vitamins and cofactors	3.728919	5
**RFX2.1.A**<br>Accelerated		
**Reactome pathway ID**	**Hypergeometric fold enrichment**	**# gene hits**
Trafficking of AMPA receptors	5.941201	4
Unblocking of NMDA receptors glutamate binding and activation	5.941201	4
**BHE22.0.D**<br>Accelerated		
**Reactome pathway ID**	**Hypergeometric fold enrichment**	**# gene hits**
Post-translational protein modification	2.155444	11
**FOXK1.0.A**<br>Accelerated		
**Reactome pathway ID**	**Hypergeometric fold enrichment**	**# gene hits**
MAPK family signaling cascades	3.24192	9
Neurotransmitter receptors and postsynaptic signal transmission	3.636769	7
Transmission across chemical synapses	3.325046	8
Neuronal system	2.501481	10
**JUN.0.A**<br>Accelerated		
**Reactome pathway ID**	**Hypergeometric fold enrichment**	**# gene hits**
Class A1 rhodopsin-like receptors	3.531506	6
**NFAC3.0.B**<br>Accelerated		
**Reactome pathway ID**	**Hypergeometric fold enrichment**	**# gene hits**
Signaling by Wnt	3.722135	8
TCF dependent signaling in response to Wnt	4.269508	6
Post-translational protein modification	1.851572	15
Cell cycle mitotic	3.320728	7
M phase	3.456268	5

**NFAC4.0.C** Accelerated		
**Reactome pathway ID**	**Hypergeometric fold enrichment**	**# gene hits**
Regulation of PTEN stability and activity	5.574468	5

**NR1H3.1.B** Accelerated		
**Reactome pathway ID**	**Hypergeometric fold enrichment**	**# gene hits**
G alpha I signalling events	3.544776	8
Fc epsilon receptor Fceri signaling	4.43097	5
Signaling by Wnt	2.962798	8
O linked glycosylation	4.43097	5
Mitotic metaphase and anaphase	3.759611	5

**SNAI1.0.C** Accelerated		
**Reactome pathway ID**	**Hypergeometric fold enrichment**	**# gene hits**
Diseases of metabolism	4.362329	6

**SOX15.0.D** Accelerated		
**Reactome pathway ID**	**Hypergeometric fold enrichment**	**# gene hits**
RUNX1 interacts with co-factors whose precise effect on RUNX1 targets is not known	5.543199	6

**STAT3.0.A** Accelerated		
**Reactome pathway ID**	**Hypergeometric fold enrichment**	**# gene hits**
Signaling by BMP	10.98011	6

**ARX.0.D** Decelerated		
**Reactome pathway ID**	**Hypergeometric fold enrichment**	**# gene hits**
Metabolism of RNA	3.275613	5

**ELK1.0.B** Decelerated		
**Reactome pathway ID**	**Hypergeometric fold enrichment**	**# gene hits**
Processing of capped intron containing pre-mRNA	4.340557	6

**HTF4.0.A** Decelerated		
**Reactome pathway ID**	**Hypergeometric fold enrichment**	**# gene hits**
Chondroitin sulfate dermatan sulfate metabolism	3.947009	9

**PBX3.1.A**		

Decelerated

Reactome pathway ID	Hypergeometric fold enrichment	# gene hits
Degradation of the extracellular matrix	4.494375	6

TBR1.0.D

Decelerated

Reactome pathway ID	Hypergeometric fold enrichment	# gene hits
Nervous system development	2.016752	9
Signaling by Robo receptors	3.004757	5

ALX1.0.B

Accelerated

Reactome pathway ID	Hypergeometric fold enrichment	# gene hits
Transcriptional regulation by RUNX1	5.884259	5
RNA polymerase II transcription	2.407197	10

Decelerated

Reactome pathway ID	Hypergeometric fold enrichment	# gene hits
Signaling by nuclear receptors	3.530556	6
RNA polymerase II transcription	1.985937	11

HXD8.0.D

Decelerated

Reactome pathway ID	Hypergeometric fold enrichment	# gene hits
RAC3 GTPase cycle	5.317778	7
RAC1 GTPase cycle	3.326178	9
RAC2 GTPase cycle	4.826218	6
Ca2 pathway	5.259341	5

BRCA1.0.D

Decelerated

Reactome pathway ID	Hypergeometric fold enrichment	# gene hits
Plasma lipoprotein remodeling	7.69196	5
Activation of the mRNA upon binding of the cap binding complex and Eifs and subsequent binding to 43S	4.615176	6

ESR1.1.A

Accelerated

Reactome pathway ID	Hypergeometric fold enrichment	# gene hits
M phase	4.553817	7
Mitotic prometaphase	4.753985	5
Cell cycle mitotic	3.09009	7

RARA.1.A

Accelerated

Reactome pathway ID	Hypergeometric fold enrichment	# gene hits
Mitotic metaphase and anaphase	6.054987	5
Cell cycle mitotic	3.798128	6

M phase	4.642157	5
Signaling by WNT	3.3967	5
Cell cycle	2.984244	6

Decelerated

**Reactome pathway ID**	**Hypergeometric fold enrichment**	**# gene hits**
RAC2 GTPase cycle	6.486301	6

RFX3.0.B

Accelerated

**Reactome pathway ID**	**Hypergeometric fold enrichment**	**# gene hits**
Trafficking of AMPA receptors	4.771018	4
Activation of NMDA receptors and postsynaptic events	3.425346	7
Assembly and cell surface presentation of NMDA receptors	5.022124	5
Neurotransmitter receptors and postsynaptic signal transmission	2.385509	10
Transmission across chemical synapses	2.223387	12
Unblocking of NMDA receptors glutamate binding and activation	5.089086	4

Supplementary Table S4. De novo motif discovery analysis results for segments predicted to be convergently accelerated

Motif name	STREME E-value	TomTom match	E-value	Database
Motif 1	0.034	STAT4	0.65	JASPAR2022 core vertebrates non-redundant v2
Motif 2	0.087	FOXD2	0.707	Jolma2013
Motif 3	0.12	SOX10	0.207	JASPAR2022 core vertebrates non-redundant v2
Motif 4	0.29	MEF2A, MEF2B, MEF2C, MEF2D	0.0386 to 0.488	JASPAR2022 core vertebrates non-redundant v2
Motif 5	0.36	KLF, SP, PATZ	2.65e-5 to 0.447	JASPAR2022 core vertebrates non-redundant v2

Supplementary Table S5. De novo motif discovery analysis results for segments predicted to be convergently decelerated

Motif name	STREME E-value	TomTom match	E-value	Database
Motif 1	3.2e-13	SOX10, FOXL1, SOX15	0.185 to 0.803	JASPAR2022 core vertebrates non-redundant v2
Motif 2	9.2e-10	SOX14, SOX21, SRY	0.181 to 0.333	Uniprobe
Motif 3	1.6e-6	STAT4	0.746	JASPAR2022 core vertebrates non-redundant v2

Motif 4	4.9e-4	KLF, SP, PATZ	4.1e-5 to 0.358	JASPAR2022 core vertebrates non-redundant v2
Motif 5	0.0025	FOXD2	0.979	Jolma 2013
Motif 6	0.015	ONECUT, CUX, SOX3	0.0148 to 0.565	JASPAR2022 core vertebrates non-redundant v2
Motif 7	0.28	-	-	-
Motif 8	0.32	MSX, BARX1	0.348 to 0.508	Jolma 2013 and Uniprobe
Motif 9	0.34	-	-	-
Motif 10	0.4	GLI, SP, KLF	0.11 to 0.931	JASPAR2022 core vertebrates non-redundant v2

Bibliography:

1. Pollard, K. S., Hubisz, M. J., Rosenbloom, K. R. & Siepel, A. Detection of nonneutral substitution rates on mammalian phylogenies. *Genome Research* **20**, 110–121 (2010).
2. Hubisz, M. J., Pollard, K. S. & Siepel, A. PHAST and RPHAST: phylogenetic analysis with space/time models. *Brief Bioinform* **12**, 41–51 (2011).
3. Saputra, E., Kowalczyk, A., Cusick, L., Clark, N. & Chikina, M. Phylogenetic Permulations: A Statistically Rigorous Approach to Measure Confidence in Associations in a Phylogenetic Context. *Molecular Biology and Evolution* **38**, 3004–3021 (2021).
4. Kulinskaya, E. On two-sided p-values for non-symmetric distributions. *arXiv:0810.2124 [math, stat]* (2008).
5. Ambrosini, G., Groux, R. & Bucher, P. PWMScan: a fast tool for scanning entire genomes with a position-specific weight matrix. *Bioinformatics* **34**, 2483–2484 (2018).
6. Kulakovskiy, I. V. *et al.* HOCOMOCO: towards a complete collection of transcription factor binding models for human and mouse via large-scale ChIP-Seq analysis. *Nucleic Acids Research* **46**, D252–D259 (2018).
7. Kostka, D., Friedrich, T., Holloway, A. K. & Pollard, K. S. motifDiverge: a model for assessing the statistical significance of gene regulatory motif divergence between two DNA sequences. *Stat Interface* **8**, 463–476 (2015).
8. Kent, W. J. *et al.* The human genome browser at UCSC. *Genome Res* **12**, 996–1006 (2002).
9. Kuhn, R. M., Haussler, D. & Kent, W. J. The UCSC genome browser and associated tools. *Brief Bioinform* **14**, 144–161 (2013).
10. Roscito, J. G. *et al.* Phenotype loss is associated with widespread divergence of the gene regulatory landscape in evolution. *Nat Commun* **9**, 4737 (2018).
11. Lawrence, M. *et al.* Software for Computing and Annotating Genomic Ranges. *PLoS Comput Biol* **9**, e1003118 (2013).
12. Quinlan, A. R. & Hall, I. M. BEDTools: a flexible suite of utilities for comparing genomic features. *Bioinformatics* **26**, 841–842 (2010).
13. Liu, C. *et al.* An ATAC-seq atlas of chromatin accessibility in mouse tissues. *Sci Data* **6**, 65 (2019).
14. McLean, C. Y. *et al.* GREAT improves functional interpretation of cis-regulatory regions. *Nat Biotechnol* **28**, 495–501 (2010).
15. Gu, Z. rGREAT: GREAT Analysis - Functional Enrichment on Genomic Regions. (2022).
16. Macosko, E. Z. *et al.* Highly Parallel Genome-wide Expression Profiling of Individual Cells Using Nanoliter Droplets. *Cell* **161**, 1202–1214 (2015).
17. Norrie, J. L. *et al.* Nucleome Dynamics during Retinal Development. *Neuron* **104**, 512-528.e11 (2019).
18. Hao, Y. *et al.* Integrated analysis of multimodal single-cell data. *Cell* **184**, 3573-3587.e29 (2021).
19. Stuart, T., Srivastava, A., Madad, S., Lareau, C. A. & Satija, R. Single-cell chromatin state analysis with Signac. *Nat Methods* **18**, 1333–1341 (2021).
20. Stuart, T. *et al.* Comprehensive Integration of Single-Cell Data. *Cell* **177**, 1888-1902.e21 (2019).
